# Interferon Regulatory Factor 4 Correlated With Immune Cells Infiltration Could Predict Prognosis for Patients With Lung Adenocarcinoma

**DOI:** 10.3389/fonc.2021.698465

**Published:** 2021-06-14

**Authors:** Xuanzong Li, Shujun Zhai, Jianbo Zhang, Dai Zhang, Shijiang Wang, Linlin Wang, Jinming Yu

**Affiliations:** ^1^ Department of Radiation Oncology, Affiliated Tumor Hospital of Xinjiang Medical University, Urumqi, China; ^2^ Department of Radiation Oncology, Shandong Cancer Hospital and Institute, Shandong First Medical University and Shandong Academy of Medical Sciences, Jinan, China; ^3^ Health Management Center, Shandong Provincial Hospital Affiliated to Shandong First Medical University, Jinan, China; ^4^ Department of Pathology, Shandong Cancer Hospital and Institute, Shandong First Medical University and Shandong Academy of Medical Sciences, Jinan, China; ^5^ Department of Radiation Oncology, School of Medicine, Shandong University, Jinan, China

**Keywords:** IRF4, tumor-infiltrating lymphocyte, prognosis, B-cells, lung adenocarcinoma

## Abstract

**Background:**

Immune related interferon regulatory factor 4 (IRF4) is a member of the IRF family, whereas the clinical significance and possible role of IRF4 in lung adenocarcinoma (LUAD) remains unclear. We aimed to investigate the role of IRF4 in predicting the prognosis of LUAD patients.

**Methods:**

Using The Cancer Genome Atlas (TCGA) database and our immunohistochemical (IHC) cohort, we analyzed the correlation between IRF4 expression and clinical characteristics, and the prognostic value of IRF4 was also evaluated in LUAD. The potential biological functions of IRF4 in LUAD were analyzed by Gene Set Enrichment Analysis (GSEA). The relationship between IRF4 and immune cell infiltration were evaluated by TISIDB database and our own IHC cohort. In addition, an immune checkpoint inhibitor (ICI) treated cohort from Gene Expression Omnibus database was used to determine the role of IRF4 in LUAD patients with immunotherapy.

**Results:**

We found that either mRNA or protein expression level of IRF4 was significantly higher in LUAD than in normal tissues (P < 0.001). The elevate in IRF4 expression in LUAD was significantly associated with the earlier clinical stage (P = 0.002). Patients with LUAD and IRF4 high expression correlated with significant longer overall survival in both TCGA database (P < 0.05) and our IHC-cohort (P = 0.001). Our results also demonstrated that IRF4 could serve as an independent favorable prognostic factor in patients with LUAD. GSEA analysis indicated that high IRF4 expression group enriched with several immune-related pathways, such as B cell receptor signaling pathway, T cell receptor signaling pathway and cytokine-cytokine receptor interaction signaling pathway. In LUAD, IRF4 positively correlated with several different immune infiltrations including various B cells, CD8+ T cells and CD4+ T cells both in mRNA and protein levels. Additionally, we found that the expression of IRF4 was positively associated with PD-1 and PD-L1 mRNA expression levels, and IRF4 high expression predicted moderate better survival in LUAD with immunotherapy (P = 0.071).

**Conclusions:**

Our results suggested that IRF4 was associated with higher B cells and T cells infiltration levels and might be a favorable prognostic biomarker in LUAD patients, whereas the potential prognostic role of IRF4 in ICI-treated patients needed further exploration.

## Introduction

Lung cancer is among the leading causes of cancer-related deaths, and it is the second frequent common cancer in the world (1, 2). Non-small-cell lung carcinoma (NSCLC) is the major type of lung cancer and has two main subtypes: Lung adenocarcinoma (LUAD) and Lung squamous cell carcinoma (LUSC) (3). Recently, immunotherapy through immune checkpoint inhibitor (ICI) has revolutionized the treatment of advanced NSCLC, and its application is extending across earlier clinical stages of NSCLC (4). However, the efficacy of ICI was shown to be varied and only a limited percentage of NSCLC patients got promising prognoses (5). The expression of PD-L1 has been served as an imperfect biomarker in NSCLC with ICI in our clinical practice (6). Besides, there is a growing appreciation on tumor microenvironment (TME), especially for a variety of tumor infiltrating lymphocytes (TILs), that may influence the efficacy of ICI (6, 7). Considering the importance of tumor immune-evasion in cancer treatment, identifying novel prognostic and immune-related biomarkers is necessary to guide clinical treatment in NSCLC.

Interferon regulatory factor 4 (IRF4) is a member to the IRF family and is specifically expressed in lymphocytes (8). As a transcription factor, IRF4 plays a major role in regulating immune responses as well as immune cell proliferation and differentiation (9). Previous studies demonstrated that abnormal expression of IRF4 identified as a diagnostic and prognostic marker was associated with various hematological malignancies such as Chronic lymphocytic leukemia (CLL), T-cell leukemia/lymphoma and Multiple myeloma (10–12). However, studies on IRF4 are scarce and the effect of IRF4 remains largely unclear in NSCLC. Using the tumor tissues from 125 NSCLC patients with surgical resection, Chen et al. described that IRF4 was an unfavorable prognostic factor in NSCLC (13). But another study demonstrated that high IRF4 expression in NSCLC patients’ peripheral blood was significantly associated with longer survival (14). Until now, there was no study focused on the specific subtypes of NSCLC to explore the prognostic role of IRF4. In this study, we aimed to investigate the role of IRF4 in predicting the prognosis of LUAD patients.

Using the genomic and clinical data from The Cancer Genome Atlas (TCGA) database, we analyzed the correlation between IRF4 mRNA expression and clinical characteristics of LUAD patients. Furthermore, both TCGA database and an untreated LUAD-cohort from our hospital were used to identify the significance of IRF4 in LUAD prognosis. The potential biological functions of IRF4 in LUAD were analyzed by Gene Set Enrichment Analysis (GSEA). In addition, the relationship between IRF4 and immune cell infiltration were evaluated. Finally, an anti-PD-1 treated LUAD cohort from Gene Expression Omnibus (GEO) database was used to determine the prognostic role of IRF4 in immunotherapy treatment. The findings in our study demonstrated that the important role of IRF4 in LUAD patients, and discovered an underlying mechanism between IRF4 and TILs correlations.

## Methods

### IRF4 Expression Analysis in TIMER and UALCAN Databases

IRF4 mRNA expression in various types of cancer was studied in the Tumor Immune Estimation Resource (TIMER) (https://cistrome.shinyapps.io/timer/) database. The TIMER database, a data mining platform targeted on the Cancer Genome Atlas (TCGA), can compare gene expression levels in different tumors (15). We also analyzed the correlation between IRF4 expression and PDCD1 (PD-1) and CD274 (PD-L1) expression in TCGA-LUAD cohort through TIMER database (16). UALCAN (http://ualcan.path.uab.edu/index.html) is a useful platform that provides graphs and plots depicting gene expression and survival curves to analyze cancer data (17). IRF4 mRNA and protein expression levels were evaluated in the UALCAN database. Furthermore, we performed a stratified analysis of IRF4 and LUAD based on patients’ age, gender and tumor stages.

### Data Collection

Gene-sequencing data and corresponding survival information of LUAD patients were obtained from TCGA database (https://portal.gdc.cancer.gov/). According to the median and 25^th^/75^th^ percentile expression value of IRF4 mRNA, LUAD patients were divided into high and low IRF4 expression groups. ICI-treated dataset (GSE93157) was obtained from Gene Expression Omnibus (GEO) database (18). GSE93157 dataset included patients with LUAD, LUSC, Head and neck squamous cell carcinoma (HNSCC), Melanoma and Skin cutaneous melanoma, and all of the patients received anti-PD-1 (pembrolizumab or nivolumab) monotherapy. Gene mRNA expression levels in the tumor samples from patients before immunotherapy was detected by the PanCancer 730-Immune Panel. We analyzed the relationship between IRF4 expression and progression free survival (PFS) in a total of 22 LUAD patients in this dataset. Basic characteristics of the TCGA and GEO data were presented in **Supplementary Tables 1** and **2**.

Between June 2016 and December 2016, a total of 73 untreated patients were included in our immunohistochemical (IHC)-LUAD cohort. All of the patients were subjected to pathologically detection and confirmed LUAD at our Hospital. The age of all patients was from 18 to 80 years, and the patients’ Eastern Cooperative Oncology Group performance status (ECOG PS) score were less than 2. Besides, clinical characteristics data and survival data for the patients from ICH-cohort were retrospectively collected. Patients mainly received surgery or chemotherapy but immunotherapy as their first line treatment. The data cutoff was December 31, 2020. The study was approved by the medical ethical committee of the Shandong Cancer Hospital and Institute.

### GSEA Analysis of IRF4 in LUAD

GSEA 4.1.0 software was used to evaluate the associations between high and low IRF4 expression subgroups and various pathway based on the entire gene expression matrix in the TCGA-LUAD cohort. In GSEA, C2.cp.kegg.v7.4.symbols.gmt was used as the reference gene set. And the IRF4 gene expression level was utilized as a phenotype label. The normalized enrichment score (NES) was calculated by performing the gene set permutations a total of 1000 times. P value < 0.05 and the false discovery rate (FDR) < 0.1 were regarded as statistical significance.

### Immune Cell Infiltration Analysis

The correlation between IRF4 and immune infiltration (B cells, CD4+ T cells and CD8+ T cells) in LUAD was analyzed by TISIDB database (http://cis.hku.hk/TISIDB/index.php) (19). TISIDB allows users to identify the role of specified gene in tumor-immune interactions through high-throughput data analysis. We also analyzed the correlation between IRF4 and CD20+ B cells, CD8+ T cells and CD4+ T cells infiltration in our own IHC-LUAD cohort.

### IHC Staining and Result Interpretation

Consecutive 4 µm thickness paraffin-embedded tumor sample sections were used for IHC staining. The IRF4 antibody (ab133590), CD8 antibody (ab93278) and CD4 antibody (ab133616) were purchased from Abcam company. The CD20 antibody (#48750) was purchased from Cell Signaling Technology company. Briefly, we put the sections in xylene to dewaxed followed by 5 minutes incubations in 100%, 95% and 75% ethanol and rehydrated in water. Then, using a high-pressure heat repair method, we finished the procedure of antigen retrieval. Subsequently, primary antibodies were used for one hour staining at 37°C, and we added HRP-labeled goat anti-rabbit secondary antibody for half-hour at 37°C. Finally, DAB was added for 60 seconds followed by counterstained hematoxylin for five minutes, and the film was sealed.

Two senior pathologists independently scored our IHC images. The proportion of cells protein expression was classified as follows: 0 point (0%, negative), 1 point (1%-10%), 2 points (11%-50%) and 3 points (51%-100%). We divided the patients into two subgroups according to the proportion of IRF4/CD4/CD8/CD20 positive cells in the samples: IRF4/CD4/CD8/CD20-low group (0-1 point) and IRF4/CD4/CD8/CD20-high group (2-3 point).

### Statistical Analysis

IBM SPSS Statistical software (version 25, USA) was used for data analysis. The difference of distribution for categorical variables in IRF4-high and IRF4-low expression subgroups were compared by the chi-square test. Survival analysis was performed by log-rank test and Kaplan-Meier method. Univariate and multivariate Cox regression model were applied to analyze the independent factors for the OS in our IHC cohort, and variables with P < 0.05 in univariate analyses were enrolled into multivariate Cox regression. P < 0.05 were considered statistically significant.

## Results

### IRF4 mRNA Expression Levels in LUAD and Other Cancers

**Figure 1:**
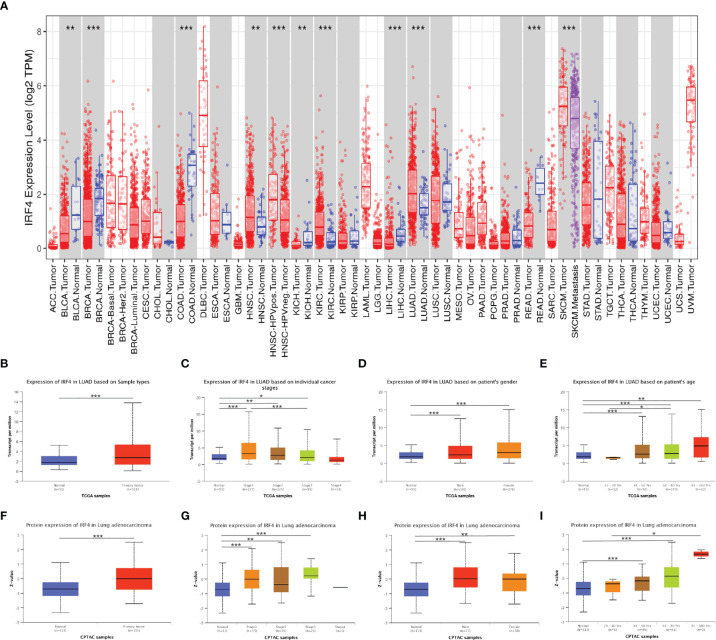
IRF4 expression levels in LUAD and other cancer types. **(A)** IRF4 expression levels in different tumor types from TCGA database were detected by TIMER. **(B–I)** Box plots showing IRF4 mRNA and protein expression levels in LUAD based on sample type, stage, gender and age by UALCAN database. (0 ≤ ***P < 0.001 ≤ **P < 0.01 ≤ *P < 0.05). LUAD, Lung adenocarcinoma.

We analyzed TIMER database to identify the differences of IRF4 mRNA expression in various tumor samples and normal samples. The IRF4 expression levels were lower in Bladder urothelial carcinoma (BLCA), Breast invasive carcinoma (BRCA), Colon adenocarcinoma (COAD), Kidney chromophobe (KICH), Liver hepatocellular carcinoma (LIHC) and Rectum adenocarcinoma (READ). On the other hand, IRF4 expression levels increased significantly in HNSCC, Kidney renal clear cell carcinoma (KIRC), and LUAD (**Figure 1A**).

Using UALCAN database, we identified that IRF4 expression was significantly higher in LUAD tumor compared to normal samples both at the transcriptional and protein levels (P < 0.001 and P < 0.001) (**Figures 1B, F**). We further analyzed IRF4 expression levels in LUAD paying attention to different clinical features including stage, gender and age. The results showed that mRNA and protein levels of IRF4 were higher in stage I-III but IV than normal samples (mRNA level: P < 0.001, P = 0.002, P =0.01; protein level: P < 0.001, P = 0.001, P < 0.001, respectively), and IRF4 mRNA level was higher in stage I than stage III in LUAD (P < 0.001) (**Figures 1C, G**). In addition, there was no significant difference between male and female in IRF4 mRNA and protein expression levels (**Figures 1D, H**). Interestingly, we found that IRF4 mRNA expression levels were lower in age from 21 to 40 years than 61 to 80 years (P =0.03). Furthermore, the protein expression levels were lower in age from 21 to 40 years than 81 to 100 years (P = 0.02) (**Figures 1E, I**).

### Prognostic Value of IRF4 in LUAD

**Figure 2:**
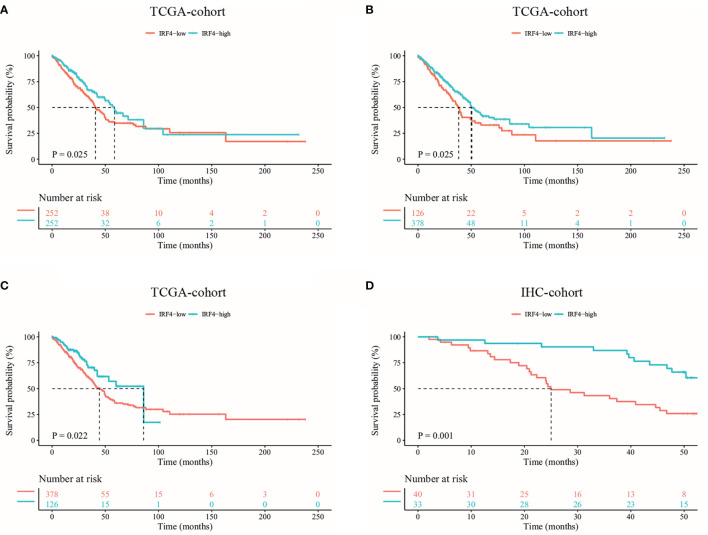
Kaplan-Meier survival curves comparing the high and low IRF4 expression in LUAD. **(A–C)** Survival curves of OS in LUAD in TCGA database (n = 504). According to the median (A) and 25^th^/75^th^ percentile **(B, C)** IRF4 mRNA expression, LUAD patients were divided into high and low IRF4 expression groups. **(D)** Survival curves of OS based on IRF4 expression in our IHC-cohort (n = 73). OS, overall survival; LUAD, Lung adenocarcinoma; IHC, immunohistochemical.

At first, we used the TCGA database to analyze the prognostic value of IRF4 mRNA expression in LUAD. Based on IRF4 expression, we divided TCGA-LUAD patients into IRF4-high and low groups relative to median expression. We found that patients in IRF4-high group were associated with better survival in TCGA-LUAD cohort (P = 0.025) (**Figure 2A**). Moreover, our finding that IRF4 high expression correlates with improved survival in LUAD are still concordant when using the thresholds of 25^th^ or 75^th^ percentile IRF4 expression levels (P = 0.025, P = 0.022, respectively) (**Figures 2B, C**).

In addition, we analyzed the relationship between IRF4 expression and survival of patients with LUAD in our IHC-cohort. A total of 73 untreated LUAD patients were included in our IHC-cohort (**Table 1**). There was no significant correlation between IRF4 and the distribution of various clinical characteristics including age, sex, smoking history and ECOG PS score in our IHC-cohort LUAD patients (P > 0.05) (**Table 2**). However, we found that low IRF4 expression was significantly associated with the clinical stage IV in LUAD patients (P = 0.002). Corresponding to the results from TCGA-LUAD database, a favorable prognosis was also found in our IHC cohort when LUAD patients with IRF4 high expression (P = 0.001) (**Figure 2D**). Based on the cox multivariate analyses, our results indicated that high expression of IRF4 was associated with the significant longer OS (HR = 1.678, 95%CI: 1.043-2.699; P = 0.033) and was an independent favorable factor for OS (HR = 1.631, 95%CI: 1.013-2.627; P = 0.044) (**Table 3**).

**Table 1:** Clinical characteristics of lung adenocarcinoma patients.

Characteristic	All patients (n = 73)
Age, n (%)	
Median (range), years	61 (34-76)
<60	35 (47.9)
≥60	38 (52.1)
Sex, n (%)	
Male	41 (56.2)
Female	32 (43.8)
Smoking history, n (%)	
Never	46 (63.0)
Ever	27 (37.0)
ECOG PS, n (%)	
0	42 (57.5)
1	31 (42.5)
Disease stage, n (%)	
I	8 (11.0)
II	10 (13.7)
III	18 (24.7)
IV	37 (50.7)
IRF4 IHC score, n (%)	
0	11 (15.1)
1	29 (39.7)
2	31 (42.5)
3	2 (2.7)

ECOG PS, Eastern Cooperative Oncology Group performance status; IHC, immunohistochemical.

**Table 2:** Clinical characteristics of lung adenocarcinoma patients with IRF4-high and low expression.

Characteristic	All	IRF4-high (n = 33)	IRF4-low (n = 40)	P
Age, n (%)				
Median (range), years	61 (34-76)	59 (42-76)	61.5 (34-72)	0.305
<60	35 (47.9)	18 (54.5)	17 (42.5)	
≥60	38 (52.1)	15 (45.5)	23 (57.5)	
Sex, n (%)				0.467
Male	41 (56.2)	17 (51.5)	24 (60.0)	
Female	32 (43.8)	16 (48.5)	16 (40.0)	
Smoking history, n (%)				0.382
Never	46 (63.0)	19 (57.6)	27 (67.5)	
Ever	27 (37.0)	14 (42.4)	13 (32.5)	
ECOG PS, n (%)				0.639
0	42 (57.5)	18 (54.5)	24 (60.0)	
1	31 (42.5)	15 (45.5)	16 (40.0)	
Disease stage				0.002
I+II+III	36 (49.3)	23 (69.7)	13 (32.5)	
IV	37 (50.7)	10 (30.3)	27 (67.5)	

ECOG PS, Eastern Cooperative Oncology Group performance status.

**Table 3:** Univariate and multivariate analyses of OS in lung adenocarcinoma patients.

Variable	OS			
	Univariate Analysis		Multivariate Analysis	
	HR (95%CI)	P	HR (95%CI)	P
Age≥60 y/<60 y	1.602 (0.823-3.118)	0.165		
Female/male	0.785 (0.407-1.515)	0.471		
Ever/never smoking	1.093 (0.562-2.124)	0.794		
Stage: IV/I-III	3.398 (1.672-6.908)	0.001	2.640 (1.264-5.514)	0.01
ECOG PS 1/0	1.254 (0.658-2.390)	0.492		
IRF4-high/low	0.309 (0.152-0.630)	0.001	0.407 (0.194-0.853)	0.017

OS, overall survival; CI, confidence interval; ECOG PS, Eastern Cooperative Oncology Group performance status; HR, hazard ratio.

### Functional Enrichment Analyses of IRF4 in LUAD

**Figure 3:**
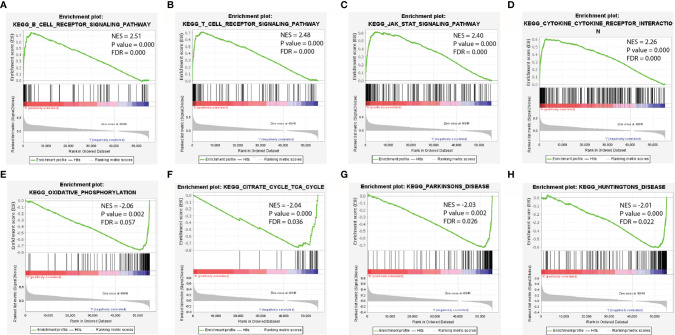
Enrichment plots from GSEA. **(A–D)** The most significant enrichment pathways with high IRF4 gene expression in LUAD. **(E–H)** The most significant enrichment pathways with low IRF4 gene expression in LUAD. GSEA, gene set enrichment analysis; LUAD, Lung adenocarcinoma.

To identify the potential mechanism related to the difference of IRF4 expression, we further predicted the most significant enrichment signaling pathways with high or low IRF4 gene expression according to the normalized enrichment score (NES) by GSEA (**Figure 3**). Our results indicated that B-cell-receptor signaling pathway (**Figure 3A**), T-cell-receptor signaling pathway (**Figure 3B**), JAK-STAT signaling pathway (**Figure 3C**) and Cytokine-cytokine-receptor-interaction (**Figure 3D**) were maximum extent enriched in IRF4 high expression subgroup. Besides, pathways including Oxidative-phosphorylation (**Figure 3E**), Citrate-cycle-tca-cycle (**Figure 3F**), Parkinsons-disease (**Figure 3G**) and Huntingtons-disease (**Figure 3H**) were maximum extent enriched in IRF4 low expression subgroup.

### Analyzing Correlation Between IRF4 and TILs in LUAD

**Figure 4:**
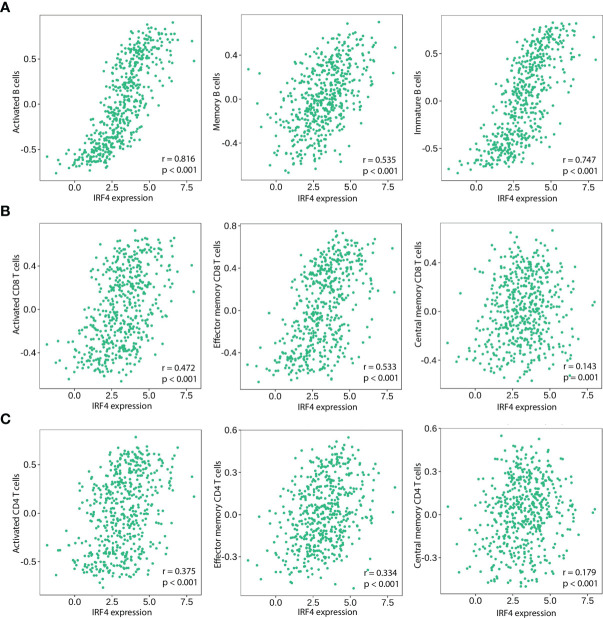
Correlation of IRF4 expression with various B and T immune cell infiltration levels in LUAD by TISIDB database (n = 517). **(A)** IRF4 expression is significantly positively correlated with activated B Cells, memory B Cells and immature B Cells in LUAD. **(B)** IRF4 expression is significantly positively correlated with activated CD8+ T Cells, effective memory CD8+ T Cells and central memory CD8+ T Cells in LUAD. **(C)** IRF4 expression is significantly positively correlated with activated CD4+ T Cells, effective memory CD4+ T Cells and central memory CD4+ T Cells in LUAD. LUAD, Lung adenocarcinoma.

Based on our GSEA results, we further assessed the relationship between IRF4 mRNA expression and tumor immune infiltrations. In LUAD, IRF4 positively correlated with infiltration of various B and T cells, including activated/memory/immature B cells (r = 0.816, P < 0.001; r = 0.535, P < 0.001; r = 0.747, P < 0.001, respectively) (**Figure 4A**), activated/effector memory/central memory CD8+ T cells (r = 0.472, P < 0.001; r = 0.533, P < 0.001; r = 0.143, P = 0.001, respectively) (**Figure 4B**) and activated/effector memory/central memory CD4+ T cells (r = 0.375, P < 0.001; r = 0.334, P < 0.001; r = 0.179, P < 0.001, respectively) (**Figure 4C**).

**Figure 5:**
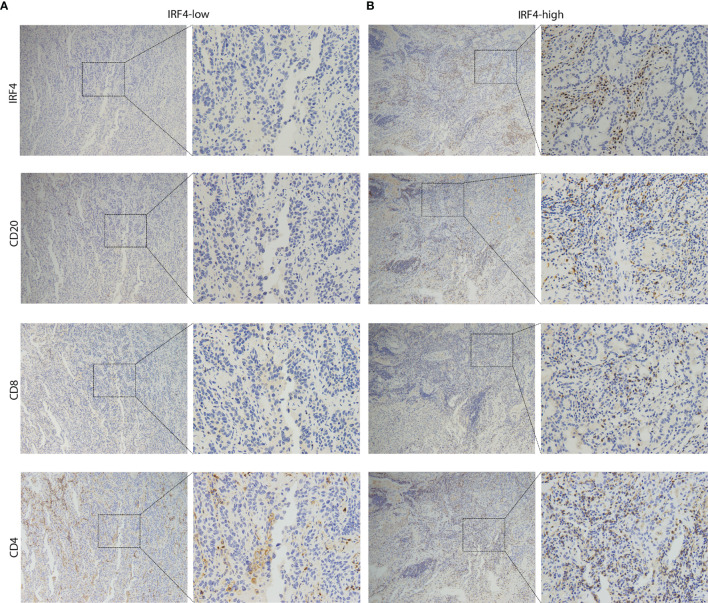
Representative IHC images of LUAD patients. **(A)** The sample from IRF4-low group was stained by IRF4, CD8, CD4 and CD20 antibodies. **(B)** The sample from IRF4-high group was stained by IRF4, CD8, CD4 and CD20 antibodies. Left panel: 10x magnification; Right panel: 40x magnification. LUAD, Lung adenocarcinoma.

Next, we used our IHC-cohort to verify the interaction between IRF4 and different immune cell subsets in protein level. We divided the patients into two subgroups according to the proportion of CD4/CD8/CD20 positive cells in the samples. Similarly, a strong correlation was found between IRF4 and all three different immune cell subsets including CD20+ B cells (P < 0.001), CD8+ T cells (P < 0.001) and CD4+ T cells (P = 0.033) in LUAD (**Table 4** and **Figure 5**).

**Table 4:** The correlation analysis between IRF4 and immune cells infiltration in lung adenocarcinoma patients.

Characteristic	IRF4-high	IRF4-low	P
	(n = 33)	(n = 40)	
CD20 IHC, n (%)			<0.001
high	31 (93.9)	11 (27.5)	
low	2 (6.1)	29 (72.5)	
CD8 IHC, n (%)			<0.001
high	28 (84.8)	8 (20.0)	
low	5 (15.2)	32 (80.0)	
CD4 IHC, n (%)			0.033
high	28 (84.8)	25 (62.5)	
low	5 (15.2)	15 (37.5)	

IHC, immunohistochemical.

### Prognostic Abilities of IRF4 in LUAD Patients With Immunotherapy

**Figure 6:**
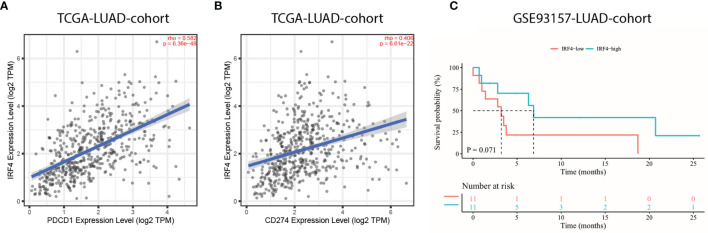
The prognostic value of IRF4 in LUAD patients with immunotherapy. **(A, B)**. Correlation of IRF4 expression with PDCD1 **(A)** and CD274 **(B)** expression in TCGA-LUAD cohort (n = 515). **(C)** Survival curves of progression free survival based on IRF4 expression in GSE93157-LUAD-cohort (n = 22). LUAD, Lung adenocarcinoma.

The introduction of ICIs, such as anti-PD-1 and anti-PD-L1, targeting on PD-1/PD-L1 pathway has revolutionized the treatment of advanced lung cancer. Therefore, the relationship between IRF4 mRNA expression and PD-1/PD-L1 mRNA expression were primarily analyzed. Importantly, the results showed that IRF4 was significantly correlated with high PDCD1 (PD-1) and CD274 (PD-L1) expression in TCGA-LUAD cohort (r = 0.582, P < 0.001; r = 0.406, P < 0.001, respectively) (**Figures 6A, B**). Nevertheless, the correlation between IRF4 and PD-L1 protein expression needed to be further validated. Then, an anti-PD-1 monotherapy cohort (GSE93157) was utilized to investigate the prognostic value of IRF4 in LUAD patients with immunotherapy. Despite IRF4-high patients correlated with moderate longer PFS contrast to LUAD patients with IRF4-low (median PFS, 6.9 months *vs.* 3.2 months, P = 0.071), the difference of PFS between IRF4-high and low subgroups failed to reveal an obvious statistical difference (**Figure 6C**).

## Discussion

In the present study, we demonstrated that either IRF4 mRNA or protein expression levels significantly increased in LUAD compared to normal samples. The findings suggested that IRF4 might be considered as a novel diagnostic biomarker in LUAD patients. Our results were comparable to a previous study conducted by Qian et al., which also revealed IRF4 was overexpressed in NSCLC (20). For the first time, using the TCGA and our own IHC cohorts, we demonstrated that IRF4 could predict favorable prognosis in LUAD. In contrast, Qian et al. demonstrated that the effect of IRF4 in LUAD cell lines was protumor through upregulating the Notch signaling pathway (20). However, a study by Wu et al. indicated that IRF4 from peripheral mononuclear cells has a protective role in advanced NSCLC patients with 77.30% adenocarcinoma and 8.51% squamous cell carcinoma (14). Taken together these controversial conclusions and our findings, we speculated that the prognostic impact on IRF4 was likely associated with the different sample sources (tumor cell or lymphocyte) in lung cancer. Considering IRF4 is specifically expressed in lymphocytes, we think that the value of its protumor effect in tumor cells is limited useful and its antitumor effect in lymphocytes is more important in clinical.

Mechanically, GSEA analysis revealed that high IRF4 expression was largely enriched in various immune-related pathways, such as B-cell-receptor signaling pathway (**Figure 3A**), T-cell-receptor signaling pathway (**Figure 3B**) and Cytokine-cytokine-receptor-interaction. Moreover, using TISDB database and our IHC-cohort, the results demonstrated that IRF4 was associated with higher infiltration of both T and B cells. In detail, we found that all three tumor infiltrating B lymphocytes (TIL-B) subsets, activated or effector memory CD8+ and activated or effector memory CD4+T cells were significantly correlated with IRF4 expression (|r| > 0.3 and P < 0.001) (**Figure 4**). In fact, several studies showed that IRF4 plays a key role in diverse pathways related immune cell including B-cell receptor signaling, T-cell receptor signaling, germinal center formation and plasma cell differentiation (21–25). Above all, high IRF4 expression might be reflect a state having predominantly tumor-specific TILs which play an important role in tumor control and prevent tumor progression (26).

Impressively, immunotherapy has revolutionized the treatment of multiple cancer types. However, up to now, finding potential biomarkers to predict the efficacy of ICIs is still challenging. Despite PD-L1 expression has been regarded as a standard biomarker to identify patients who may benefit from ICIs treatment, additional biomarkers including different TILs are being investigated to further improve the benefit of these patients. Unlike the well-investigated T-cells in tumor infiltration, the role of TIL-B has been scarcely studied in different cancers. Recently, a series of studies indicated that TIL-B was involved in responses of patients to immunotherapy, demonstrating the crucial role of B cells in cancer treatments (27–29). Ku et al. conducted a study aiming to identify the prognostic effect of various TILs in NSCLC patients with PD-1 inhibitor treatment, and demonstrated that intratumoral B cells density was positively correlated with the favorable OS (30). Otherwise, several studies indicated that the tumor mutational burden (TMB) could be used as a potential biomarker for the efficacy of ICI across multiple cancer types (31, 32). However, some studies also suggested that the positive association between TMB and the efficacy of immunotherapy not always existed (33–35). For instance, wang et al. demonstrated that LUAD patients with STK11 mutations correlated with higher TMB but worse prognosis after immunotherapy, partly due to the less immune cell infiltration or PD-L1 low expression (33). In our study, we found that IRF4 high expression represented as a hot immune environment in the LUAD along with both TIL-B and T cells high infiltration levels. Besides, GSEA analysis indicated that these TIL-B and T cells might be at an active state in the IRF4-high LUAD patients. In addition, we also demonstrated that IRF4 was positively correlated with PD-1 and PD-L1 expression levels based on TCGA-LUAD data analysis. However, considering the important role of PD-L1 expression in immunotherapy, the correlation between IRF4 and PD-L1 protein expression needs to be further investigated. Though an overall statistically beneficial effect of IRF4 was not found in our anti-PD-1 monotherapy cohort, the trends suggested that it could bring clinical benefit in LUAD patients with immunotherapy.

Undeniably, some limitations were existed in our study. In our IHC-cohort, some tissues were obtained from biopsy, which might potentially bias the results. Additionally, only 22 LUAD patients received anti-PD-1 treatment in our immunotherapy cohort, and the small samples limited any solid conclusions.

Collectively, our study demonstrated a close association between IRF4 and immune infiltration, and IRF4 could be used as a prognostic marker in patients with LUAD. Nevertheless, further clinical studies are required to validate our findings, and largescale ICI-related studies are needed to investigate the role of IRF4 in LUAD patients with immunotherapy.

## Data Availability Statement

The datasets presented in this study can be found in online repositories. The names of the repository/repositories and accession number(s) can be found in the article/**Supplementary Material**

## Ethics Statement

The studies involving human participants were reviewed and approved by Shandong cancer hospital. Written informed consent for participation was not required for this study in accordance with the national legislation and the institutional requirements.

## Author Contributions

LW and JY contributed to the study concept and design, and critical revision of the manuscript for important intellectual content. XL performed the data analysis and drafted the manuscript. XL, SZ, JZ, DZ and SW contributed to data collection and interpretation. All authors contributed to the article and approved the submitted version.

## Funding

This work was partially supported by funds from The National Key Research and Development Projects of China (2018YFC1312201), Radiation Oncology Innovate Unit, Chinese Academy of Medical Sciences (2019RU071), the Academic Promotion Program of Shandong First Medical University (2019ZL002) and the foundation of National Natural Science Foundation of China (81972863, 81627901 and 82030082).

## Conflict of Interest

The authors declare that the research was conducted in the absence of any commercial or financial relationships that could be construed as a potential conflict of interest.
