# Supplementary material for: Interferon Regulatory Factor 4 Correlated With Immune Cells Infiltration Could Predict Prognosis for Patients With Lung Adenocarcinoma
Source: Front Oncol. 2021 Jun 14;11:698465. doi: 10.3389/fonc.2021.698465 (PMC8236722; doi:10.3389/fonc.2021.698465)
Supplement: Supplementary file 17 [file Table_2.docx]

Supplementary Material

# Supplementary Figures and Tables

## Supplementary Table 1 Basic characteristics of the TCGA data included in the study.

| Characteristic | TCGA-LUAD  (n = 504) |
| --- | --- |
| Age (years), n (%)  ≤60  >60  Unknown  Gender, n (%)  Male  Female  Race, n (%)  White  Black  Asian  American Indian  Unknown  Smoking history, n (%)  Yes  No  Unknown  Disease stage  I  II  III  IV  Unknown | 157 (31.2)  337 (66.9)  10 (1.9)  234 (46.4)  270 (53.6)  387 (76.8)  52 (10.3)  7 (1.4)  1 (0.2)  57 (11.3)  419 (83.1)  71 (14.1)  14 (2.8)  270 (53.6)  119 (23.6)  81 (16.1)  26 (5.2)  8 (1.6) |

## Supplementary Table 2 Basic characteristics of the GEO data included in the study.

| Characteristic | GSE93157-LUAD  (n = 22) |
| --- | --- |
| Age, n (%)  Median (range), years  <60  ≥60  Sex, n (%)  Male  Female  Smoking history, n (%)  Former  Current  Never  ECOG score  0  1  Previous lines  0  1  ≥2  Drug  Pembrolizumab  Nivolumab | 58 (42-79)  12 (54.5)  10 (45.5)  15 (68.2)  7 (31.8)  11 (50.0)  8 (36.4)  3 (13.6)  5 (22.7)  17 (77.3)  4 (18.2)  8 (36.4)  10 (45.5)  8 (36.4)  14 (63.6) |
